# Automated Analysis of the Two-Minute Walk Test in Clinical Practice Using Accelerometer Data

**DOI:** 10.3390/brainsci11111507

**Published:** 2021-11-13

**Authors:** Katrin Trentzsch, Benjamin Melzer, Heidi Stölzer-Hutsch, Rocco Haase, Paul Bartscht, Paul Meyer, Tjalf Ziemssen

**Affiliations:** 1 Center of Clinical Neuroscience, Neurological Clinic, University Hospital Carl Gustav Carus, TU Dresden, Fetscherstr. 74, 01307 Dresden, Germany; katrin.trentzsch@uniklinikum-dresden.de (K.T.); benjamin.melzer@outlook.com (B.M.); Heidi.Stoelzer-Hutsch@uniklinikum-dresden.de (H.S.-H.); Rocco.Haase@uniklinikum-dresden.de (R.H.); paul@bartscht.name (P.B.); paul.meyer.15@web.de (P.M.); 2 Institute of Biomedical Engineering, TU Dresden, Fetscherstr. 29, 01307 Dresden, Germany

**Keywords:** multiple sclerosis, gait analysis, mobility, digital tools and applications

## Abstract

One of the core problems for people with multiple sclerosis (pwMS) is the impairment of their ability to walk, which can be severely restrictive in everyday life. Therefore, monitoring of ambulatory function is of great importance to be able to effectively counteract disease progression. An extensive gait analysis, such as the Dresden protocol for multidimensional walking assessment, covers several facets of walking impairment including a 2-min walk test, in which the distance taken by the patient in two minutes is measured by an odometer. Using this approach, it is questionable how precise the measuring methods are at recording the distance traveled. In this project, we investigate whether the current measurement can be replaced by a digital measurement method based on accelerometers (six Opal sensors from the Mobility Lab system) that are attached to the patient’s body. We developed two algorithms using these data and compared the validity of these approaches using the results from 2-min walk tests from 562 pwMS that were collected with a gold-standard odometer. In 48.4% of pwMS, we detected an average relative measurement error of less than 5%, while results from 25.8% of the pwMS showed a relative measurement error of up to 10%. The algorithm had difficulties correctly calculating the walking distances in another 25.8% of pwMS; these results showed a measurement error of more than 20%. A main reason for this moderate performance was the variety of pathologically altered gait patterns in pwMS that may complicate the step detection. Overall, both algorithms achieved favorable levels of agreement (r = 0.884 and r = 0.980) with the odometer. Finally, we present suggestions for improvement of the measurement system to be implemented in the future.

## 1. Introduction

Multiple sclerosis (MS) is a chronic inflammatory, progressive disease of the central nervous system. Based on its multifocal, inflammatory lesions in the central nervous system, MS is characterized by deficits in different neurological functional systems, which leads to a wide range of symptoms and a highly individualized course of the disease [1,2]. It is important to phenotype the different symptoms of MS to adapt the management of the disease [3,4]. For many people with MS (pwMS) the limitation on the ability to walk is a clinical hallmark of their disease. Walking problems have a major impact on important areas of life and contribute significantly to the patient’s quality of life. Up to 85% of pwMS report impairments in their ability to walk [5]. Kister et al. stated that 5 years after disease onset, 45% of pwMS reported mild gait deficits, and after 30 years of disease, only 18% of pwMS were able to walk without problems or with minimal limitations [6]. Different pathophysiological components such as spasticity, paresis, or sensitivity and balance disorders contribute to the development of patient-specific gait disorders [7,8].

In pwMS, walking impairments are characterized by a decreased gait speed, walking endurance, step rate, and cadence in addition to an increased variability of gait [9,10,11]. All these gait impairments increase with the progression of the disease. In routine clinical practice, limitations in mobility are primarily assessed with the Expanded Disability Status Scale (EDSS). Less frequently, various time-based walking tests are applied, which are often subject to intraindividual and interindividual variation [12,13,14]. For a better detection of mobility impairments and a high-quality, clinically relevant characterization of pwMS, an objective multimodal assessment of gait changes, such as the Dresden protocol for multidimensional walking assessment (DMWA), is important [15,16,17]. Different walking domains, such as gait quality, maximal walking speed, patient-reported outcomes, and also gait endurance should be assessed, with the aim to provide a more objective and standardized measurement of walking ability in addition to the EDSS [16].

Specifically, testing of walking endurance is used as an important marker in various medical settings. The Cooper 12-min walk test was originally developed for physical fitness [18]. As time progressed, shorter versions of this endurance walk test, such as the six- and two-minute walk test (6MWT, 2MWT) were developed [19]. In medicine, the gold standard of endurance testing is considered to be the 6MWT [7]. However, some patients are unable to walk for longer than two minutes. So, the 6MWT is often too strenuous and time consuming for cardiac patients and also for pwMS, so the 2MWT became a practical alternative in this case [19,20,21]. This is a popular and well-established walking test to obtain a detailed impression of walking ability [22] that can be well compared to the 6MWT [20,22,23].

For subtle changes in gait and the detection of an early deterioration in endurance, an accurate measurement of the distance covered is required. Estimating the total distance by multiplying the number of gaits covered does not meet the requirement for accuracy. Unlike an estimate of the total distance travelled, an odometer objectively measures the distance travelled. An odometer is clinically approved and considered the gold standard. Odometers are in principle well suited for measuring longer distances with quite high measuring accuracy [24]. For this reason, they are widely used for the measurement of walking distance in clinical environments [16,25,26,27,28]. An odometer is a measuring wheel with an integrated counter, a handpiece, and a digital display. It is designed for distance measurements on flat ground. For this purpose, the odometer is pushed over the floor in such a way that the wheel rolls permanently without slip. Although an odometer objectively measures the distance travelled, it does not measure the exact distance travelled by the patient within the given time. Using a stopwatch, the tester must measure exactly two minutes so that the odometer can be stopped afterwards, which creates a delay effect of the distance measurement. Patients can show different evasive movements when walking the distance. This is due, among other things, to the pathological gait pattern and obstacles in the course. Furthermore, the turn at the end of the gait is displayed inaccurately because the reversal angle with the odometer is different. In addition, there is the possibility of a speed influence that is subconsciously transferred from the tester with the odometer to the person being tested. To prevent a lower inter-rater reliability by changing the respective examiner, the person to be tested should walk the 2MWT completely alone.

The aim of the study is to address exactly this problem by improving the existing monitoring of multidimensional gait analysis in its complexity and efficiency and increasing its objectivity. A digitalization in this field, through the integration of appropriate algorithms can optimize the efficiency and quality of patient management [29]. The use of inertial measurement units (IMUs) is becoming increasingly popular for determining gait deficits, such as with the 6MWT [30]. In particular, the distance traveled is often calculated from IMU data [31,32,33]. Retory et al. compared the distance traveled, which was classically calculated from the product of the number of steps (from video recordings) with the median step length, with IMU data from an accelerometer and a correlation of r = 0.99 [32] was shown. When calculating the distance traveled using IMUs no other measurement instruments were needed, thus resulting in less bias in the measuring method. Furthermore, the use of IMUs facilitated the application of the 2MWT in a setting outside the clinic. The development of such a digital measurement method is relevant to the structural shift towards home-based assistive devices for which simple digital measurement methods are needed, as well as to simplify the clinical measurement process while improving measurement accuracy.

For this purpose, we developed two algorithms using accelerometer data. These two approaches were compared and their basic functionality was evaluated in a monocentric study with the gold standard of odometers.

## 2. Materials and Methods

This work investigates whether the 2MWT with an odometer can be replaced by a 2MWT with accelerometers. From the sensor data of the body-worn sensors, the total distance can be determined using two developed algorithms. The first approach provides an overall evaluation of the total acceleration (Digiwalk algorithm, DWA) signals. The second approach calculates the total distance based on an average stride length (Mobility Lab algorithm, MLA). The primary focus of this study is to compare the accuracy of the DWA and MLA with the gold-standard odometer.

### 2.1. Population

Data from 562 patients were used for the analysis, which were recorded between July 2018 and February 2020 at the MS Center Dresden of University Hospital Carl Gustav Carus, Dresden. All pwMS completed a multidimensional gait analysis according to the DMWA protocol as part of their clinical outpatient visit. We included patients with a reliable diagnosis of MS, which were able to walk with or without assistive devices. Each participant was examined according to good clinical practice guidelines. The study was approved by the local ethics committee (BO-EK-320062021).

### 2.2. Procedure of the 2-Min Walk Test (2MWT)

For the 2MWT, the pwMS wore six Mobility Lab Opal sensors (APDM, Portland, OR, USA) and were asked to walk along a hospital corridor approximately 25 m long for two minutes. Walkers could be used during the test, but the patient had to be able to walk independently. Short breaks could also be taken, but these were recorded during the two minutes. To allow accurate distance measurement, the examiner walked behind the patient to match the patient’s speed and not dictate his or her own walking pace. The distance traveled was recorded with an odometer, and the respective time needed was checked with a stopwatch. For each patient, the covered distance of the 2MWT was measured with the odometer and the file with the acceleration values was archived.

### 2.3. Distance Measurement with Acceleration Sensors

**Figure 1:**
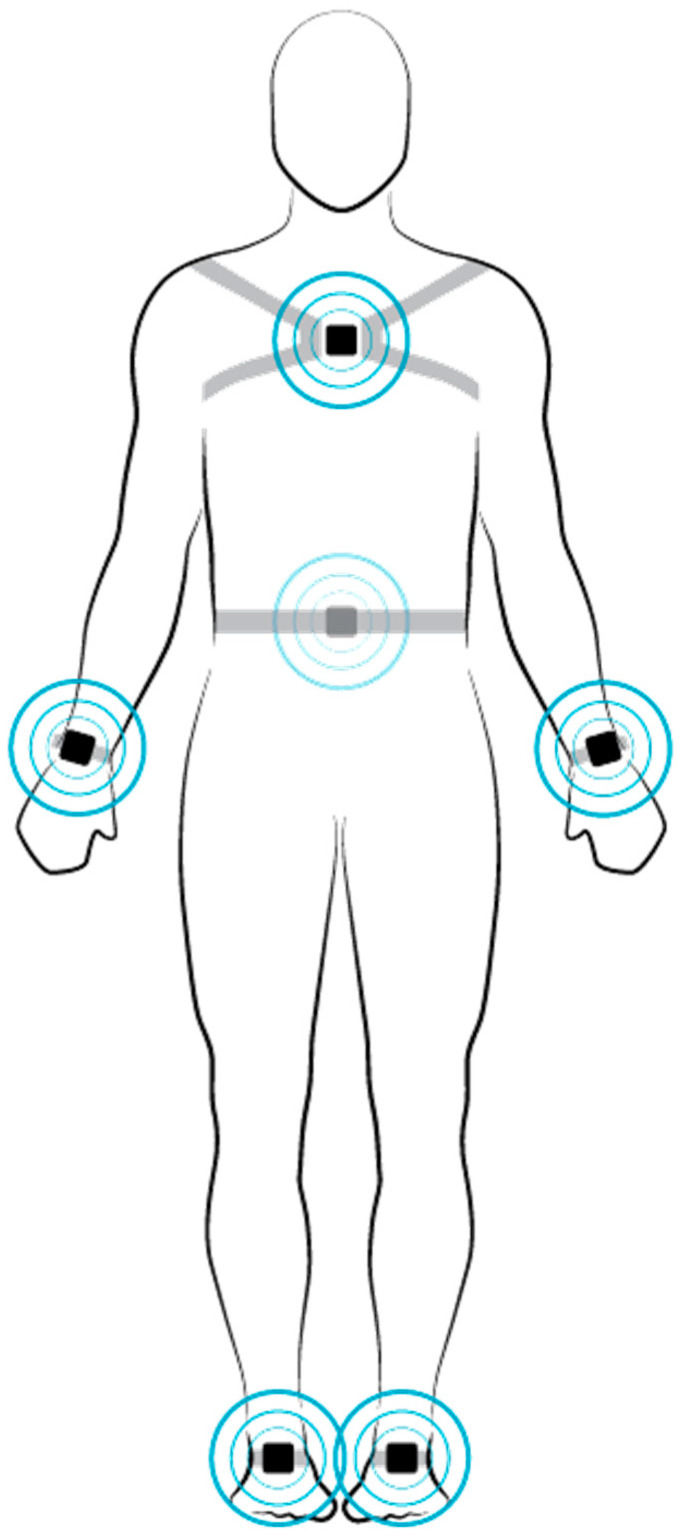
Demonstration of the six Mobility Lab sensors (APDM, Portland, OR, United States), both sensors applied to the forefoot serve as the basis for the calculation.

An accelerometer is a sensor that determines the acceleration it experiences by measuring the inertial force acting on a test mass. In the accelerometer-based measurement method, we used Mobility Lab Opal sensors (APDM, Portland, OR, USA) to measure spatiotemporal gait parameters of patients during walking trials. There were six individual sensor units that were attached to the patient’s wrists, ankles, sternum, and lower back (Figure 1) [34].

Location of the motion sensors on specific body parts was used to obtain valid gait and balance parameters. Being consistent with other motion sensors worn on the body, the sensor to measure upper sway was placed in front of the sternum 2 cm below the jugular fossa [35]. Another sensor was placed on the lumbar spine at L5 to measure lower trunk balance [35,36,37]. To measure the arm swing, two sensors were placed at the left and right wrist, 4 cm from the dorsum of the hand [38]. The last two sensors for spatiotemporal gait parameters were attached to the forefoot [39,40]. Each sensor unit contained a three-axis accelerometer, three-axis gyroscope, and a magnetometer. Measurement data were collected after each walking test by plugging the sensors into the access point, which automatically generated a single file containing the raw kinematic measurements [41]. We used the acceleration data contained in these files to estimate the distance walked by the patient.

While accelerometers are a well-established method for gait analysis in the clinical environment, they are currently not commonly used for measuring walking distances [42]. However, acceleration measurements of any sensor unit carry a measurement error caused by tiny drift rates of the gyroscopes that must be compensated for by the algorithms employed [43]. In fact, extracting precise distance values from acceleration data requires exceptionally sophisticated algorithms that employ methods from the field of inertial navigation [43].

There are essentially two different algorithmic approaches to extract distance information from acceleration data. 

#### 2.3.1. Digiwalk Algorithm (DWA)

The underlying idea of the DWA is to calculate velocity from acceleration (a(t)) by integration over time. Repeating this procedure to integrate velocity over time results in the distance traveled (s(t)) at the following rate:

$$s\left(t\right)=\iint^{\;}a\left(t\right)d^{2}t$$

**Figure 2:**
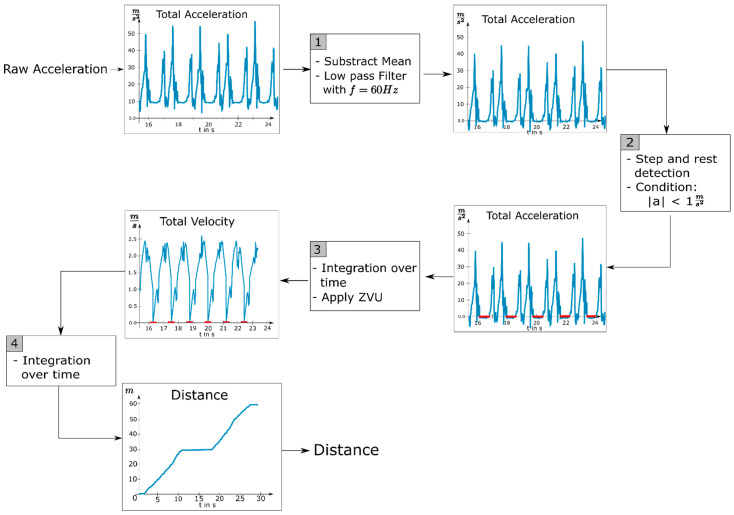
Flowchart for determining the distance walked; ZVU = zero velocity updates.

Foxlin and Bebek et al. demonstrated that two sensors that were each attached to an ankle provided sufficient information about the distance walked [43,44]. The reason being that the ankles experienced the greatest acceleration during walking which led to a high signal to noise ratio and a highly detectable movement signal. Furthermore, we leveraged the fact that each foot stands completely still for a brief moment during each walking cycle. Velocity and distance can be calculated from acceleration data by integration over time. Due to the drift of the acceleration sensors and the resulting double integration of a possible error, the calculated values deviated more from the real values over longer distances. To compensate for these errors, the periodical standstills of the feet allowed us to continuously set the velocity to zero whenever a resting foot was detected, which eliminated the drift of the calculated values. We followed the approach of Foxlin who proposed to apply these zero velocity updates (ZVU) to solve the problem of drifting values for velocity and distance [43]. To the extent that this method builds on the raw acceleration data from the Mobility Lab system, we called this approach the Digiwalk algorithm. The following section describes the DWA that we implemented to determine the distance traveled from the raw acceleration data. Figure 2 illustrates the process.

**Figure 3:**
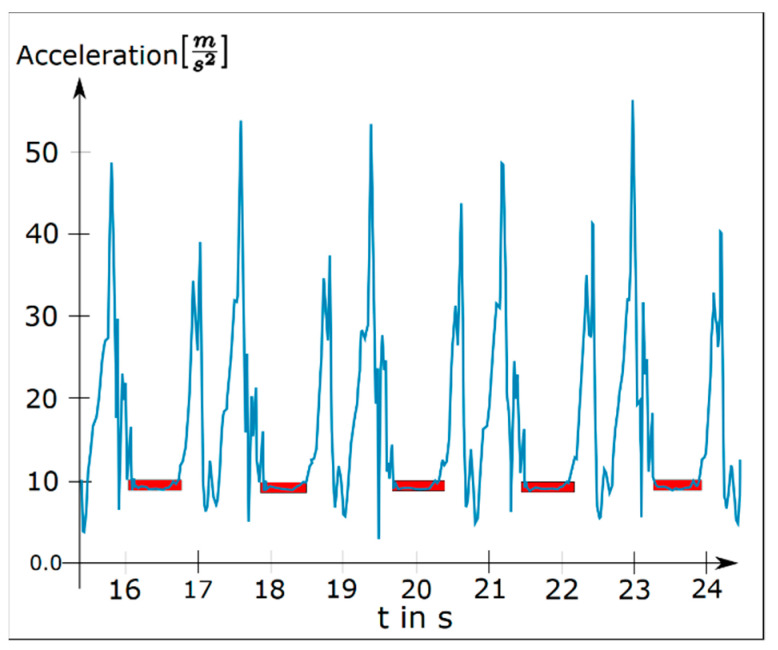
Total acceleration of one foot with detected resting periods (red).

The first step was to preprocess and clean the acceleration data from the influence of gravity. Therefore, from each acceleration component $a_{x}$, $a_{y}$, and $a_{z}$, we subtracted its mean. Subsequently we reduced unwanted frequency components in the signals (which were sampled at 128 Hz) by applying a low pass filter with a cut-off frequency of 60 Hz. Next, we detected the time periods during which each foot was resting. Since a resting foot should only experience the acceleration of gravity, which we removed from the signal, we searched for sections with a total acceleration of zero plus a threshold for measurement inaccuracy. We determined the optimal threshold in preliminary experiments to be 1 $\frac{m}{s^{2}}$ by varying the threshold value until each step of a test subject was detected for an appropriate duration (stance duration). The results of our step detection are illustrated in Figure 3, which shows the raw acceleration data of one foot as well as the time intervals in which the algorithm declared the foot was resting.

**Figure 4:**
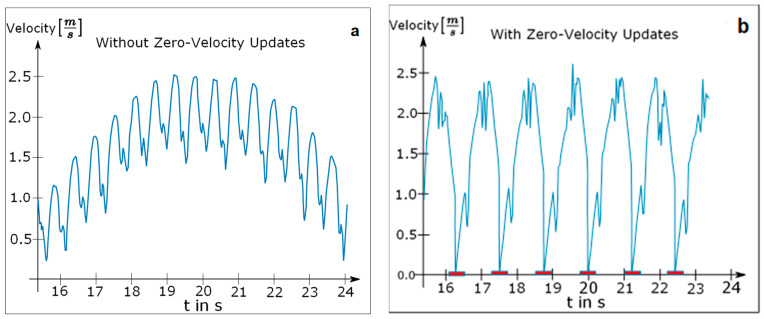
(**a**) Velocity curve of a single foot without zero velocity updates; (**b**) velocity curve of a single foot with zero velocity updates.

In addition to the detection of a single resting foot, we also identified time periods during which the patient completely stopped walking. This occurred frequently during the walking tests since some patients were severely restricted in their ability to walk for longer periods of time. The patient was declared to be resting as soon as both feet were found to be resting simultaneously for more than one second. The integration process was paused for both feet as soon as the standstill of the patient was detected. To calculate the distance walked, we separately integrated each component of the acceleration signal twice over time. Integration was suspended for the time interval in which either one or both feet were detected to be resting and continued afterwards starting with an initial value of zero. The difference between the calculated velocity curves of one foot with and without ZVU can be seen in Figure 4.

Even though each step was clearly visible in the velocity diagram without ZVU in Figure 4a, the curve contradictorily suggests that the foot never reached a velocity of zero. By contrast, the velocity curve with applied ZVU in Figure 4b depicts each step realistically with short periods of zero velocity while the foot is resting.

**Figure 5:**
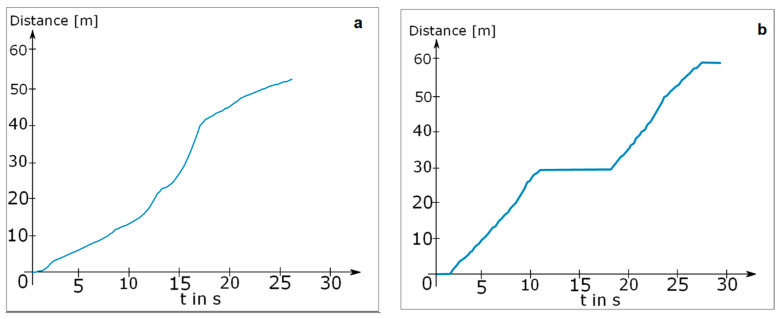
Acceleration of one foot with (**a**) calculated distance without rest detection; (**b**) calculated distance with rest detection.

Finally, the difference between the calculated distances with and without stop detection is shown in Figure 5, which depicts an experiment in which a test subject walked to stop at t = 10 s for approximately 6 s. While the distance calculated without stop detection continued growing even though the test subject was standing still, the curve with stop detection stopped growing properly at this point.

#### 2.3.2. Mobility Lab Algorithm (MLA)

Capela et al. presented a possible approach for inertial navigation [45]. They used raw accelerometer data processing for the 6MWT in a clinical setting and employed an activity detection to distinguish walking from standing times. By multiplying the average stride lengths by the number of steps taken, the walked distance was determined [45]. The number of steps could be recorded using a pedometer or peak identification of the acceleration data. In our work, we used the same approach as just described. The distance traveled in two minutes were estimated by multiplying the stride length by the number of steps per minute. As the calculation was based on the stride length output by the Mobility Lab System, we refer to it as the Mobility Lab algorithm.

### 2.4. Statistical Methods

Continuous variables were presented as mean ± standard deviation (SD) or median with interquartile range (IQR), where appropriate. Categorical variables were described in absolute numbers and percentages. We calculated the mean of the differences between the DWA, MLA, and odometer measurement series, the SD, and the 95% limits of agreement (=mean ± 1.96 × SD) to describe the agreement between the MLA and the DWA to the standardized odometer measurement method. Bland–Altman plots, Pearson’s r, and intraclass correlation coefficients (ICC) were calculated to compare the three estimates of distance (odometer, DWA, MLA). ICC levels were interpreted according to the guidelines of Koo and Li (below 0.50: poor, between 0.50 and 0.75: moderate, between 0.75 and 0.90: good, above 0.90: excellent) [46]. Mean values were tested for significance using a *t*-test for systematic differentiation between the distance traveled by the different measurement methods and the use of assistive devices. In order to assess important variables influencing the measurement series and their measurement errors, Kendall’s tau-b correlation analyses were performed. Kendall’s tau-b (τb) has been defined for level 0.1 to 0.3 as weak, for level 0.3 to 0.5 as moderate, and above 0.5 as strong correlation. Linear model analyses were performed to determine variables influencing DWA and MLA. An identity linkage function linear model were used for normally distributed data with the factors of age, sex, degree of disability (via EDSS), the use of assistive devices, and subtype of MS (relapsing–remitting, primary progressive, and secondary progressive). The level of statistical significance was set at α = 5%. All statistical analyses were performed using IBM SPSS Statistics 27 (SPSS, Chicago, IL, USA).

## 3. Results

The analysis used data from 562 pwMS performing the 2MWT. There was an age distribution of 16 to 79 years among the patients, with a mean age of 43.15 (SD ± 12.31) years. A figure of 69.8% of the pwMS were female and the disease duration of patients averaged 8.57 (SD ± 7.51) years. An overview of patient characteristics is shown in Table 1.

**Figure 6:**
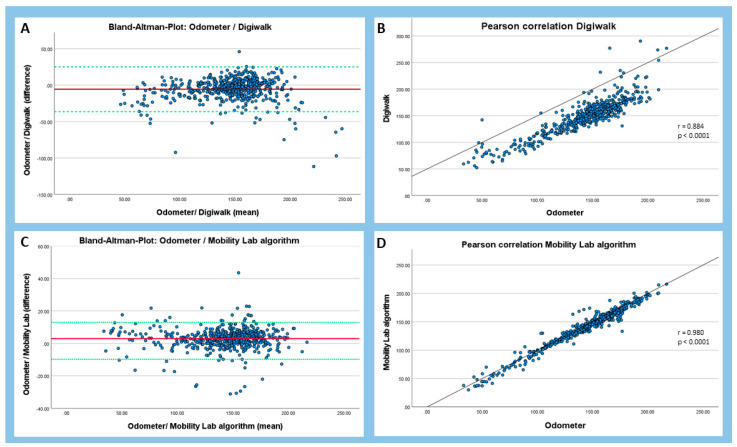
Bland–Altman and Pearson’s correlation plots. (**A**) Bland–Altman plot of the Digiwalk measurement; (**B**) Pearson correlation of the Digiwalk measurement; (**C**) Bland–Altman plot of the Mobility Lab algorithm; (**D**) Pearson correlation of the Mobility Lab algorithm. The red line shows the mean of the differences between the two respective methods, and the dashed green horizontal lines show the upper and lower 95% limits of agreement (=mean ± 1.96 × SD). R = correlation coefficient.

With the comparison of the respective mean values of the two measuring methods to the odometer it was shown that the DWA overestimated the values (149.20 ± 32.33) whereas the MLA, calculated using cadence and average step length, underestimated the covered distance (140.6 ± 32.58) compared with the distance measured by the odometer (143.52 ± 32.57) (Table 1, Figure 6).

Bland–Altman plots revealed upper and lower limits of agreement of 24.10 and −36.40 for the DWA and upper and lower limits of agreement of 15.68 and −9.84 for the MLA (Figure 6). The two algorithms showed good to excellent correlations with the odometer (DWA: r = 0.884, ICC = 0.871; MLA: r = 0.980, ICC = 0.976). 

**Figure 7:**
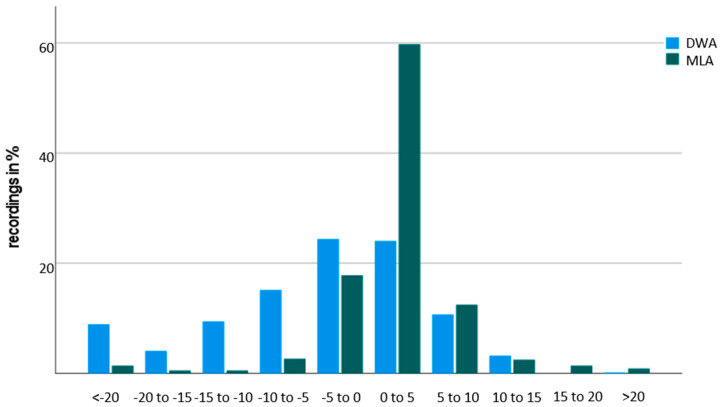
Histogram of the relative frequencies of relative measurement errors for the Digiwalk algorithm (DWA) and the Mobility Lab algorithm (MLA) compared to the current gold-standard odometer.

The relative measurement error was calculated for both algorithms, and the distribution is visualized in Figure 7. Most of the recordings showed a relatively small measurement error of 10% or less. The mean measurement error was 9.21 ± 14.7% for the DWA and 4.06 ± 4.61% for the MLA, respectively.

Comparing the covered distance between the DWA and the odometer, 272 data sets had a relative measurement error of less than 5%, which was 48.4% of the total number of data sets. However, 145 (25.8%) data sets remained with a measurement error of up to 10% and 25.8% had a measurement error of more than 20% error. 

In the second approach, comparing the MLA and the odometer, a relative measurement error of less than 5% was present in 436 (77.6%) data sets. Another 85 data sets (15.1%) showed a measurement error of less than 10% and the remaining 7.3% had over 20% error.

**Figure 8:**
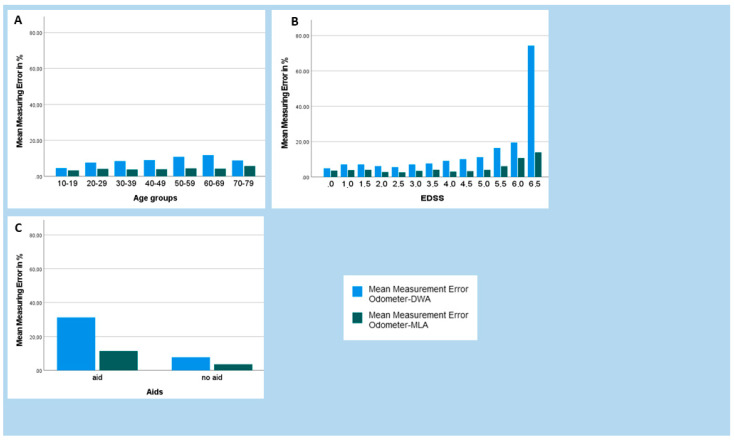
Representation of the relative measurement errors of the Digiwalk algorithm (DWA) and the Mobility Lab algorithm (MLA) in relation to the influence variables of age (**A**), Expanded Disability Status Scale (**B**), and use of aids (**C**).

The relative measurement errors were analyzed in relation to influencing variables such as age, degree of disability, and the use of assistive devices and are shown in Figure 8.

When age (Figure 8A) of the pwMS was considered, the MLA measurement errors appeared to be independent of age. For the DWA, there was a peak in measurement error at the age of 60 to 69.

Measurement errors tended to increase with increasing disability slightly earlier with the DWA (EDSS 4.0) than with the MLA (EDSS 5.5) (Figure 8B). 

The relative measurement errors also increased with the use of assistive devices, especially with DWA. The highest relative measurement error that occurred was 31.27% when measuring with the DWA with aids. In contrast, the lowest relative measurement error of 3.57% occurred when measuring with the MLA without aids (Figure 8C). The mean difference between the odometer and the DWA using assistive devices was −14.44 ± 16.39 m, compared to without assistive devices −5.10 ± 15.44 m (*p* = 0.001). Compared to the DWA, the mean difference between the odometer and the MLA was lower with aids 4.82 ± 7.28 m and without aids 2.79 ± 6.45 m (*p* = 0.074) (Table 2).

The measurement error of the DWA showed a weak correlation with age (τb = 0.0173), the use of aids (−0.159), and disease disability (Table 3), as well as a moderate correlation with double support (τb = 0.0359). There were no such correlations for the MLA.

In a multifactorial linear model approach, level of disease disability was solely associated with the measurement error of the DWA (T = 6.395; *p* < 0.001; CI 95% 0.184 to 0.348), whereas disease disability (T = −3.464; *p* = 0.001; CI 95% −0.086 to −0.024) and age (T = 2.329; *p* = 0.02; CI 95% 0.003 to 0.039) where associated with the measurement errors of the MLA. Sex, the use of walking aids, and the subtype of MS were not associated with any algorithm in the respective linear model.

## 4. Discussion

Walk endurance tests are important to quantify walking parameters accurately [47,48]. Therefore, we applied two novel algorithm–based approaches using accelerator sensors in comparison to the current standard measurement using the odometer in the walking assessment of pwMS. Our results demonstrated that the DWA achieved a good performance (measurement error < 5%) in about half of the pwMS tested (48.4%) and the MLA in considerably more pwMS (77.6%). Overall, both algorithms achieved favorable levels of agreement (DWA: ICC = 0.871; MLA: ICC = 0.976) with the odometer. These results are comparable to other calculations based on accelerometers for measuring distance traveled [49].

Sensor data for the assessment of mobility in MS have gained interest over recent years. Creagh et al. demonstrated how signal-based features related to movement can be extracted from sensors in smartphones and smartwatches and showed good correlations with clinical outcomes [47]. Karle et al. performed initial approaches of a 2MWT outside a clinical environment for pwMS. For this, the average cadences were processed from the raw acceleration data of an activity monitor. An average cadence between a clinical environment and an outside environment was compared [48]. Unfortunately, both studies did not include a gold standard of measurement to verify the respective measurement system. Our approach including two algorithms aimed for the digitalization of walking assessment in the neurological practice through the integration of appropriate algorithms that could optimize patient management. While we demonstrated overall performances of good to excellent in the two algorithms in comparison to the gold standard, measurement errors in some subgroups of pwMS increased. It was reasonable to assume that the measurement error of a distance measurement with accelerometers was highly dependent on the gait pattern of the person. We investigated how accurately an irregular gait pattern affected accelerometer measurements. Thus, we found a robust association between disease disability of the pwMS and measurement error for both algorithms (MLA and DWA). Furthermore, there may have been an inherent error in the calculated relative measurement error because the measurement accuracy of the odometer was unknown.

For the DWA, there was a moderate correlation (τ = 0.359) between double support and the measurement error. It should be taken into account that only six gait parameters (cadence, stride length, double support, gait speed, lateral step variability, and number of turns) were considered in our analyses. Nevertheless, there are many more spatiotemporal gait parameters that can be tested with respect to measurement error. For example, the number of breaks taken was not considered in our work. It should be noted that some pwMS, especially in older age or with increased degree of disability, were not able to perform the 2MWT continuously but needed short standing breaks in between.

Other measurement inaccuracies can also result from the rotation of the subjects at the turning point of the measurement course. An algorithm modification should be implemented for this. There must be an intelligent threshold for detecting the rotation. El-Gohary et al. proposed a threshold of ±5°/s as the beginning and end of each rotation [50]. For general testing with differently impaired subjects, these thresholds need to be evaluated. Cheng et al. have already provided reliable algorithms for detecting rotations and rotation speeds. Similar processing algorithms can be implemented in the DWA in the future. More accurate detection of rotations will increase the accuracy of the total distance [51].

The DWA measurement error was more often positive than negative, whereas this seemed to be the other way round for the MLA. Positive error values mean that a distance value derived from the accelerometers was higher than the reference value. This in turn suggests that the DWA more often failed to detect steps, rather than misclassifying a signal segment as a step when there was none. In particular, the DWA was found to have difficulties to some degree in accurately detecting steps once the gait pattern deviated heavily from the norm. For this reason, the DWA tended to miss steps frequently in some pwMS, resulting in the acceleration not resetting correctly to zero. As a result, acceleration, velocity, and distance increased incorrectly. Additionally, the accurate detection of resting phases, as well as the differentiation between resting phases, slow walking, and turning, proved to be difficult. Any misjudgment of the person’s current state led to acceleration errors, which in turn led to a rapidly growing error in the calculated distance. 

In conclusion, the quality of the measurement was highly dependent on successful step detection, which in turn depended on the gait pattern being as regular as possible. Possible solutions to these problems are discussed in the next section.

## 5. Future

In the future, we will improve our analytical algorithm described here by adopting the approach of Foxlin, who implemented a measurement system to track the position of a walking person [43]. Since the distance walked can be calculated from the difference in position between two points in time, this approach would also be suitable for our approach. Foxlin tracks the position using calculations based on a complex sensor fusion of accelerometers and gyroscopes. The calculation errors of velocity, distance, and attitude originating from sensor drifts are compensated by only navigating in an open loop manner during the strides phase of each foot. Zero velocity updates are not directly applied to the velocity measurements by resetting it to zero but are fed into an extended Kalman filter (EKF) as pseudo measurements. The EKF corrects the state components acceleration, velocity, distance, attitude, and angular velocity after each measurement, reducing the position error that previously grew cubically in time to an error growing linearly with the number of steps taken. In this manner, the position drift that occurs during each stride phase is corrected by monitoring the correlation between velocity and position error. The performance of this measurement system was evaluated by indoor experiments in which a person walked for 322 s, covering 118.5 m, resulting in a position error of only 0.3%. It is however unclear, exactly how well this algorithm will perform on pathologically altered gait patterns, especially since the calculations also rely on detecting the stance of the phases of the feet. 

A completely different approach would be the use of Bluetooth beacons which could eliminate the problem of accurately detecting the stance phases of the feet that has proven to be the main problem. Therefore, this approach could be tested in the future. Since the main weakness of this method is the position accuracy, further tests have to show how a position error of about 1 to 2 m affects the calculated distance.

Applying artificial intelligence is another possibility and is a promising approach. As we have shown, machine learning algorithms enable the integration and visualization of a wide variety of gait parameters in routine clinical practice [52]. Thus, model calculations could be performed based on the available spatiotemporal gait data to predict the distance traveled. For this, further studies are needed to determine the necessary input data and the most useful (combination of) sensor systems.

## Figures and Tables

**Table 1:** Characterization of people with multiple sclerosis (MS) presented as mean [mean] with standard deviation [SD] or median with interquartile range (IQR); MS = multiple sclerosis; RRMS = relapsing–remitting MS; PPMS = primary progressive MS; SPMS = secondary progressive MS; EDSS = Expanded Disability Status Scale; 2-min walk = 2MWT.

		pwMS (*n* = 562)
Mean age (years; mean ± SD)		43.15 ± 12.31
Females (N, %)		392 (69.8%)
Disease duration (years; mean ± SD)		8.57 ± 7.51
MS Subtype		
	RRMS (N, %)	490 (87.2%)
	PPMS (N, %)	55 (9.8%)
	SPMS (N, %)	13 (3.0%)
EDSS (median, IQR)		2.5 (1.5–3.5)
Aids		
	with	35 (6.2%)
	without	527 (93.8%)
2MWT		
	2MWT with odometer in m (mean, SD)	143.52 ± 32.57
	2MWT with Digiwalk in m (mean, SD)	149.20 ± 32.33
	2MWT with MobiLab in m (mean, SD)	140.61 ± 32.58

**Table 2:** Comparison of the measurement series with the aids (mean ± SD) by t-tests; 2MWT = two-minute walk test; OM = odometer; DWA = Digiwalk algorithm; MLA = Mobility Lab algorithm.

	With Aids	Without Aids	*p*
2MWT OM	70.98 ± 22.89	148.34 ± 29.91	<0.001
2MWT DWA	85.42 ± 16.55	153.44 ± 28.44	<0.001
2MWT ML	66.16 ± 23.19	145.55 ± 26.53	<0.001
Difference OM-DWA	−14.44 ± 16.39	−5.10 ± 15.44	0.001
Difference OM-MLA	4.82 ± 7.28	2.79 ± 6.45	0.074

**Table 3:** Kendall´s tau-b correlation between demographic data, clinical outcomes, and parameter of gait (n = 562); EDSS = Expanded Disability Status Scale; DWA = Digiwalk algorithm; MLA= Mobility Lab algorithm.

			Measurement Error DWA	Measurement Error MLA
Age		τ	0.173 **	0.091 **
Sex		τ	0.041	−0.007
Aids		τ	−0.159 **	0.147
Disease Duration		τ	0.073 *	0.001
Disease Disability (EDSS)		τ	0.241 **	−0.029
Parameter of gait				
	Cadence	τ	−0.116 **	0.155 **
	Stride Length	τ	−0.191 **	0.119 **
	Double Support	τ	0.359 **	−0.010
	Gait speed	τ	−0.184 **	0.143 **
	Lateral Step variability	τ	0.177 **	0.075 **
	Number of turns	τ	−0.164 **	0.051

* *p* < 0.05, ** *p* < 0.01. EDSS = Expanded Disability Status Scale, DWA = Digiwalk algorithm; MLA = Mobility Lab algorithm.

## Data Availability

The data presented in this study are available on request from the corresponding author. The data are not publicly available due to patient confidentiality.
